# Supplementary material for: Characterization of the Binding Modes of Cu2+ Ions with Tyrosine and Ado, AMP, ADP, and ATP: A Comprehensive Potentiometric, Spectroscopic, and Computational Approach
Source: Int J Mol Sci. 2025 Sep 11;26(18):8865. doi: 10.3390/ijms26188865 (PMC12469315; doi:10.3390/ijms26188865)
Supplement: Supplementary file 1 [file ijms-26-08865-s001.zip › ijms-3827327-supplementary.pdf]

SUPPORTING INFORMATION

Characterization of the Binding Modes of Cu^{2+} Ions with Tyrosine and Ado, AMP, ADP, and ATP: A Comprehensive Potentiometric, Spectroscopic, and Computational Approach

Patrycja Sadowska, Romualda Bregier-Jarzębowska, Wojciech Jankowski, Mateusz Gołdyn, Renata Jastrząb*

Faculty of Chemistry, Adam Mickiewicz University in Poznań, Uniwersytetu Poznańskiego 8, 61-614 Poznań, Poland;

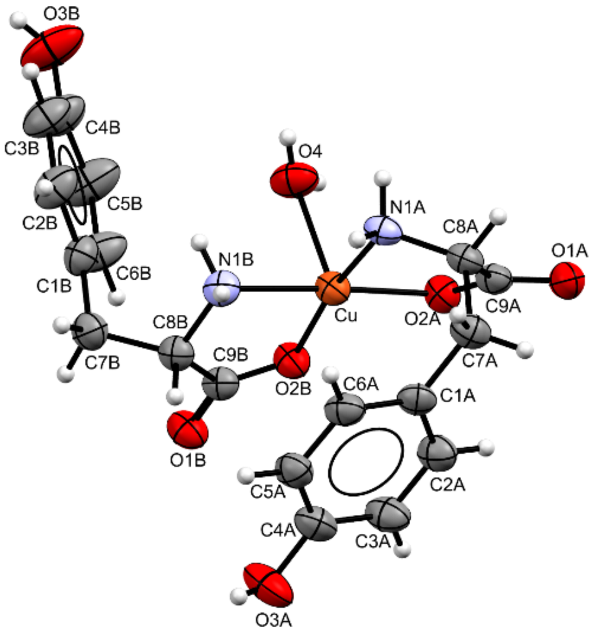

Figure S1. An ORTEP representation of the asymmetric unit of $[CuH_2(Tyr)_2(H_2O)]\times 1.5H_2O$ with an atomic numbering scheme. Thermal ellipsoids were plotted at the 50% probability level. Solvent molecules were omitted due to the solvent mask used.

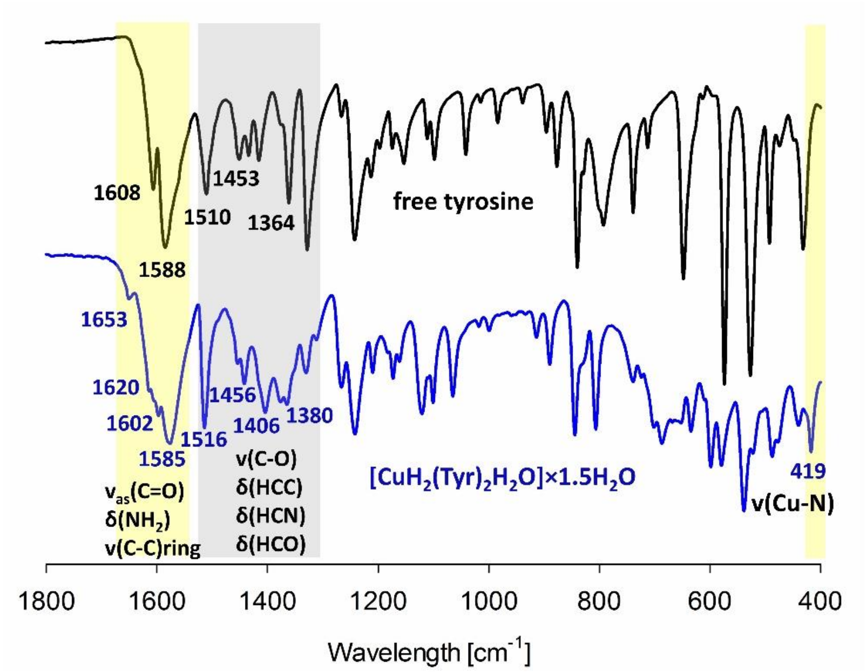

Figure S2. A comparison of the FT-IR spectra of free tyrosine and the $[CuH_2(Tyr)_2(H_2O)]\times 1.5H_2O$ crystals obtained in their solid state.

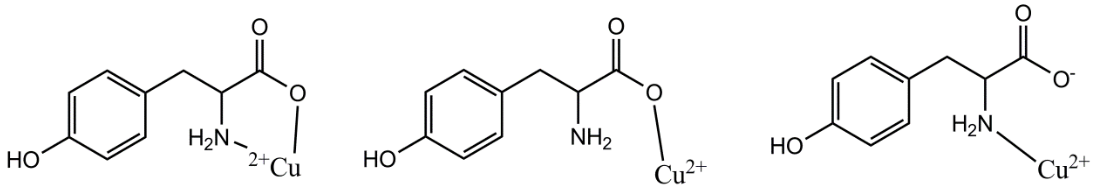

CuH(Tyr)_1 **CuH(Tyr)_2** **CuH(Tyr)_3**

Figure S3. Schemes of possible interactions between H(Tyr) and a copper(II) ion.

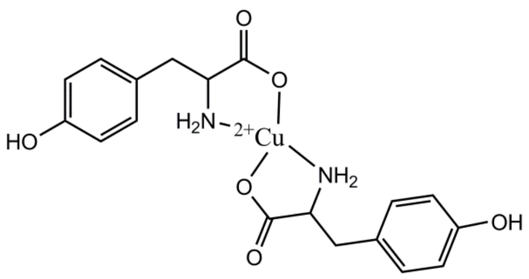

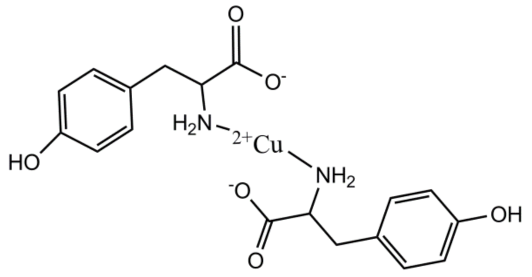

$CuH_2(Tyr)_2$_1 **$CuH_2(Tyr)_2$_2**

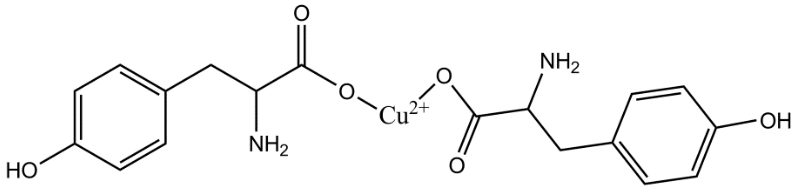

$CuH_2(Tyr)_2$_3

Figure S4. Schemes of possible interactions between two H(Tyr) molecules and a copper(II) ion.

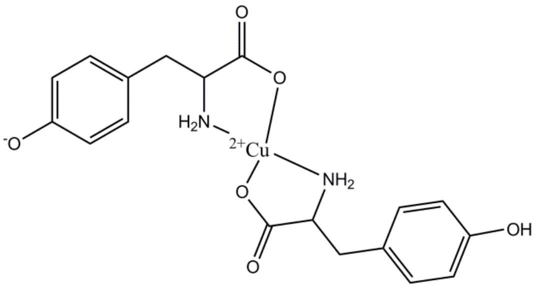

CuH(Tyr)$_2$_1

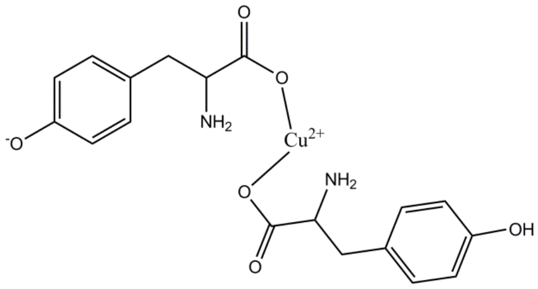

CuH(Tyr)$_2$_2

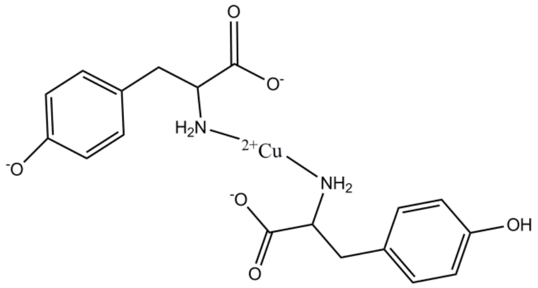

CuH(Tyr)$_2$_3

Figure S5. Schemes of possible interactions between H(Tyr) and Tyr molecules and a copper(II) ion.

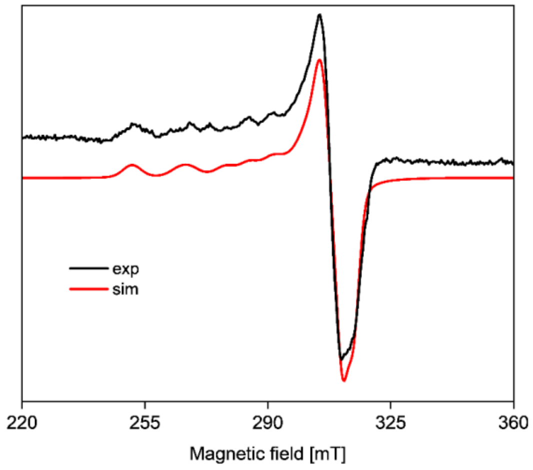

Figure S6. The experimental and simulated EPR spectra for the Cu(Tyr)H(AMP) complex at a pH = 6.6; $C_{Cu^{2+}=L=L'}=1\times10^{-3}$ M.

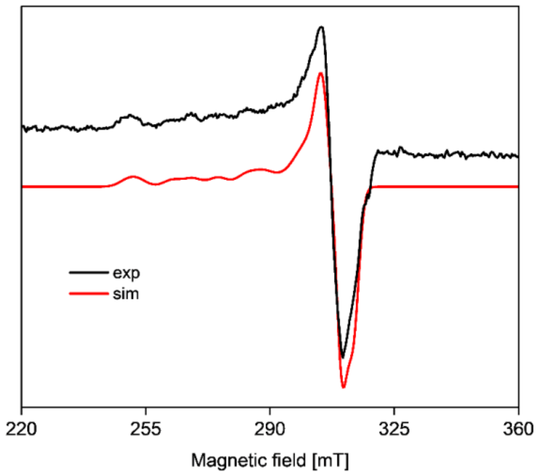

Figure S7. The experimental and simulated EPR spectra for Cu(Tyr)H(ADP) at a pH = 5.6; $C_{Cu^{2+}=L=L'}$=1×10^{-3} M.

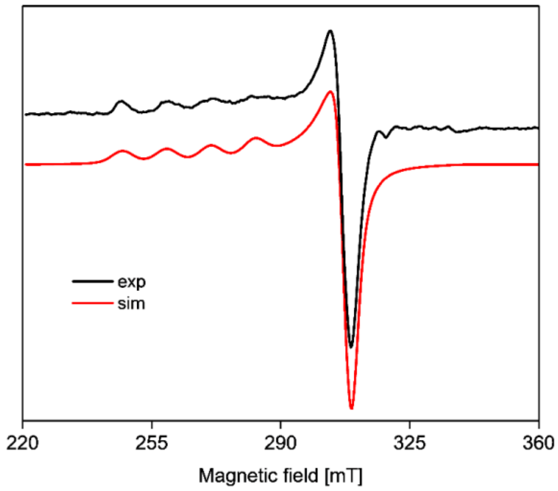

Figure S8. The experimental and simulated EPR spectra for the Cu(Tyr)H$_4$(ATP) complex at a pH = 2.5; $C_{Cu^{2+}=L=L'}$=1×10^{-3} M.

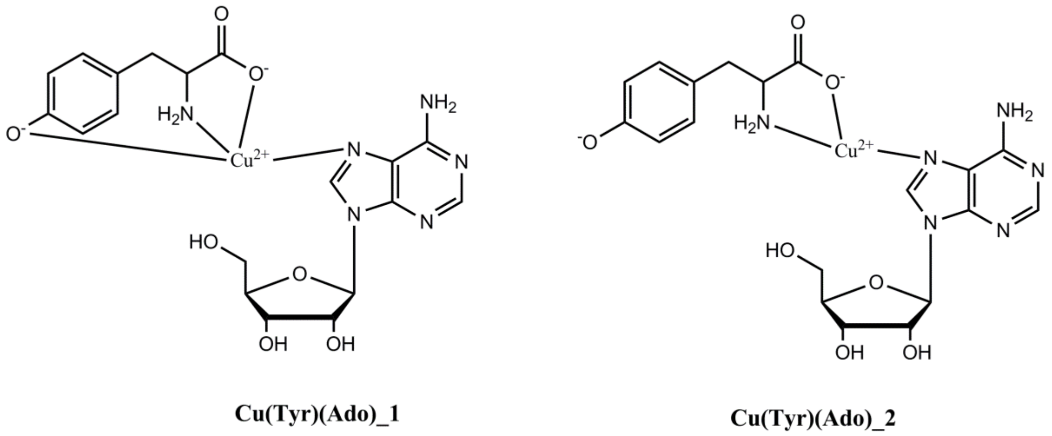

Figure S9. Schemes of possible interactions between Tyr and Ado and copper(II).

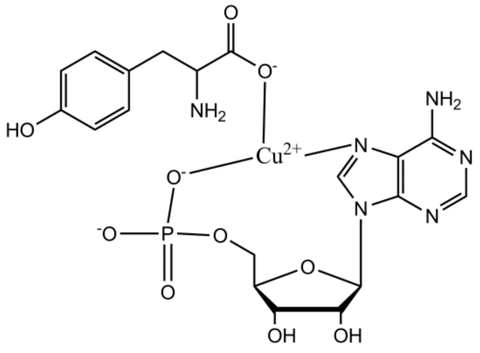

Cu(Tyr)H(AMP)$^{-}$_1

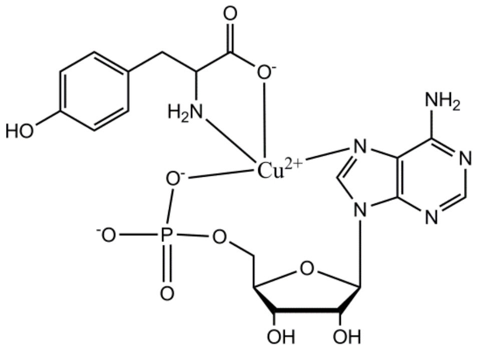

Cu(Tyr)H(AMP)$^{-}$_2

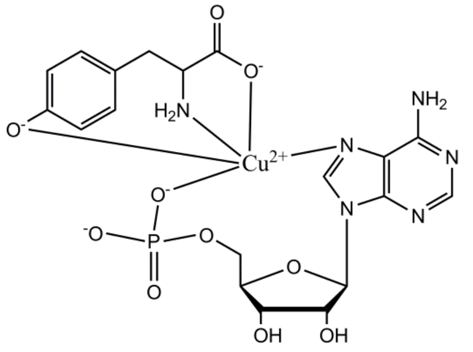

Cu(Tyr)(AMP)$^{2-}$_1

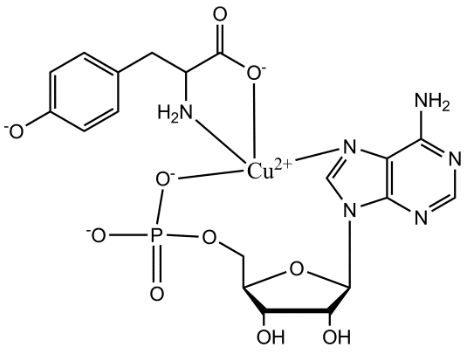

Cu(Tyr)(AMP)$^{2-}$_2

Figure S10. Schemes of possible interactions between H(Tyr) or Tyr and one molecule from AMP and copper(II).

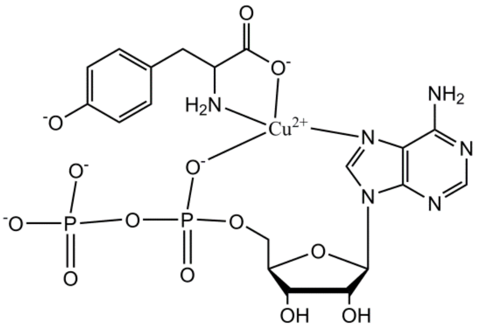

Cu(Tyr)(ADP)$^{3-}$_1

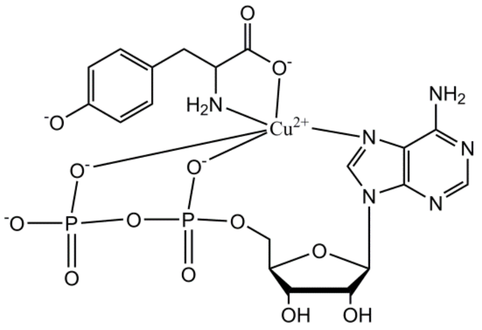

Cu(Tyr)(ADP)$^{3-}$_2

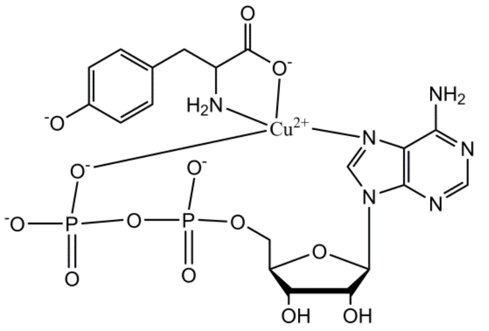

Cu(Tyr)(ADP)$^{3-}$_3

Figure S11. Schemes of possible interactions between Tyr and ADP molecules and copper(II).

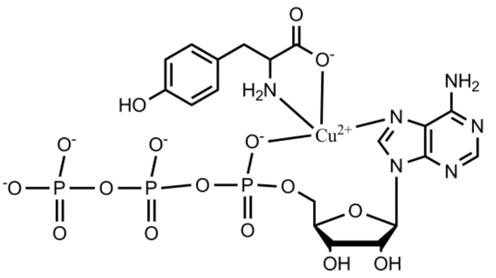

Cu(Tyr)H(ATP)$^{3-}$_1

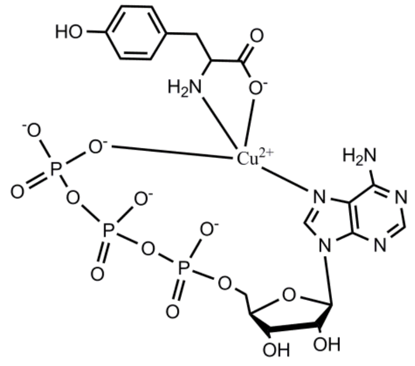

Cu(Tyr)H(ATP)$^{3-}$_2

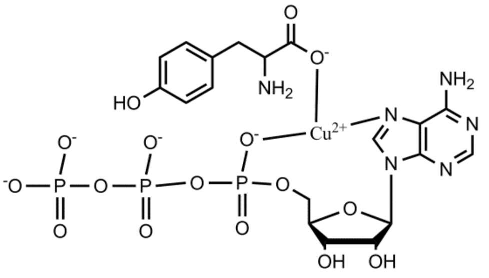

Cu(Tyr)H(ATP)$^{3-}$_3

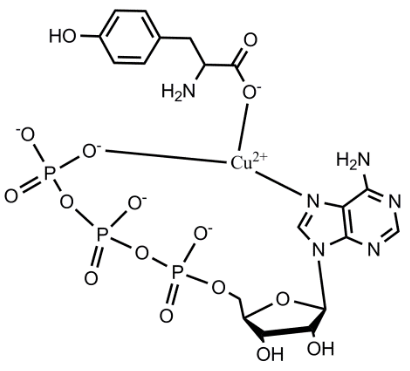

Cu(Tyr)H(ATP)$^{3-}$_4

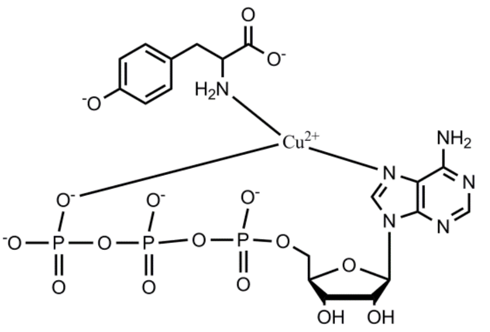

Cu(Tyr)(ATP)$^{4-}$_1

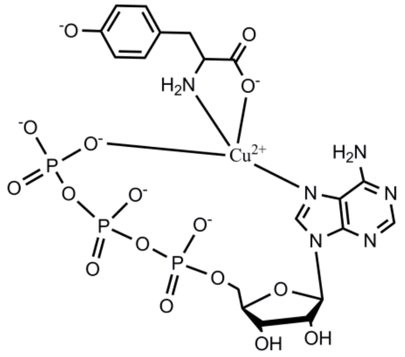

Cu(Tyr)(ATP)$^{4-}$_2

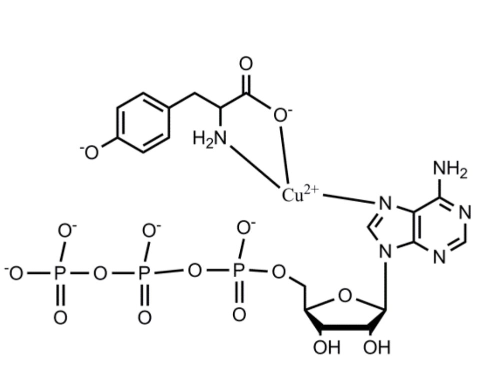

Cu(Tyr)(ATP)$^{4-}$_3

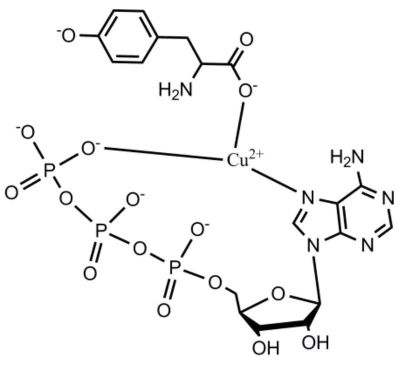

Cu(Tyr)(ATP)$^{4-}$_4

Figure S12. Schemes of possible interactions between H(Tyr) or Tyr and ATP and copper(II).

Table S1. Crystal data and structure refinement for the $[CuH_2(Tyr)_2(H_2O)]\times1.5H_2O$ complex.

Complex	$[CuH_2(Tyr)_2(H_2O)]\times1.5H_2O$
Empirical formula	$C_{18}H_{22}CuN_2O_7\times1.5H_2O$
Formula weight/g·mol^{-1}	468.94
Crystal system	monoclinic
Space group	$P2_1$
a/Å	12.0133(2)
b/Å	6.0351(1)
c/Å	14.9473(2)
α/°	90
β/°	102.395(1)
γ/°	90
V/Å^3	1058.44(3)
Z, Z′	2, 1
ρ_{calc}/g·cm^{-3}	1.471
μ/mm^{-1}	1.896
F(000)	488.0
Crystal size/mm^3	0.45×0.12×0.03
Radiation/Å	Cu Kα (λ=1.54184)
2θ range/°	6.054 to 152.58
Index ranges	$-14\leq h\leq15$<br>$-7\leq k\leq7$<br>$-17\leq l\leq18$
Reflections collected	12411
Independent reflections	4359 [R_{int}=0.0381, R_{sigma}=0.0376]
Reflections with I≥2σ (I)	4210
Data/restraints/parameters	4359/1/256
Final R indexes [I≥2σ (I)]	R_1=0.0279, wR_2=0.0714
Final R indexes (all data)	R_1=0.0293, wR_2=0.0725
Goodness-of-fit on F^2	1.028
Largest diff. peak/hole/e·Å^{-3}	0.18/-0.22
Flack parameter	-0.048(16)
CCDC deposit no.	2417185

Table S2. Calculated energies, sums of monomers, interaction energies, and Gibbs free energy of possible H(Tyr) complexes with copper(II) ion in aqueous solution.

Interaction scheme of H(Tyr)–Cu(II) complex	Energy [Hartree]	Sum of monomers [Hartree]	Interaction energy [kcal/mol]	ΔG [Hartree]
Cu_H_(Tyr)	-826.159693	-825.878229	-176.6	-826.016549

Table S3. Atomic coordinates of optimized structures of H(Tyr) complex with copper(II) ion.

Cu_H_(Tyr)			
Atom	x	y	z
Cu	21.0384193788	-12.8657234213	1.4894396108
C	14.7782513351	-12.3809452548	0.439538231
C	14.3971929339	-13.4202622866	-0.4201490341
C	15.2272393524	-13.8053723523	-1.4864660639
C	16.4439978342	-13.1429657726	-1.6812793369
C	16.8467672551	-12.0978022816	-0.830064257
C	15.9978524159	-11.7280730938	0.229631975
C	18.1752211713	-11.4078482283	-1.0389046791
C	19.3521216206	-12.3172892138	-0.6524023929
C	20.7025795225	-11.6294120821	-0.8722179074
O	13.1781995260	-14.0298344463	-0.1669353567
N	19.2509951579	-12.7963448443	0.774153316
O	21.6294305206	-11.8457787730	0.0808310004
O	20.9490137685	-10.9401838593	-1.8713403718
H	14.1173812276	-12.0983290308	1.2525135839
H	14.9230547435	-14.6064950633	-2.1557756967
H	17.0811372137	-13.4350253716	-2.5139401858
H	16.2842255330	-10.9118589312	0.8899709676
H	18.3208717145	-11.1321744262	-2.0897450364
H	18.2274201294	-10.4801366814	-0.4544062451
H	19.3475480307	-13.2091046073	-1.2905729195
H	12.9701508364	-14.7423181363	-0.8018558196
H	18.7162698126	-12.1368868718	1.3529303197
H	18.7976489657	-13.7138449701	0.8340462985

Table S4. Calculated energies, sums of monomers, interaction energies, and Gibbs free energy of possible complexes of two H(Tyr) molecules with copper(II) ion in aqueous solution.

Interaction scheme of two H(Tyr)–Cu(II) complexes	Energy [Hartree]	Sum of monomers [Hartree]	Interaction energy [kcal/mol]	ΔG [Hartree]
Cu_H$_2$_(Tyr)$_2$_2	-1455.38662164	-1454.95432357	-271.2709296077024	-1455.076121

Table S5. Atomic coordinates of optimized structure of complex consisting of two H(Tyr) and copper(II) ions.

	Cu_H2_(Tyr)2		
Atom	x	y	z
Cu	15.7710849128	-7.7143913185	-1.9923686921
C	9.6561101908	-6.3139477691	-0.5513869158
C	9.4728945149	-7.7056893636	-0.5141245583
C	10.5620637694	-8.5625057504	-0.3000598255
C	11.8410456253	-8.0140440181	-0.1267455591
C	12.0471538362	-6.6241619797	-0.1610548647
C	10.9365416522	-5.7843356987	-0.3767286466
C	13.4307324168	-6.0408691911	-0.0140767988
C	14.0248084109	-5.599878018	-1.3729673868
C	15.4589462743	-5.1126078598	-1.124242441
O	8.1784127529	-8.1708004571	-0.6934656334
N	14.063200357	-6.7526304983	-2.3127945328
O	16.4031739824	-6.0162099091	-1.3272299742
O	15.6637273451	-3.9529298738	-0.6962231755
C	20.9222580341	-11.0736701348	0.6348825961
C	21.9806944788	-10.3062946268	0.1239884424
C	21.9028282444	-9.7532129646	-1.1620937821
C	20.7524428179	-9.9715154779	-1.9346200712
C	19.6802111108	-10.7339753862	-1.4434480799
C	19.7842295563	-11.2803558578	-0.1485627462
C	18.4263842344	-10.9448084484	-2.2586007334
C	17.2453447625	-10.1285719808	-1.71185293
C	15.9302142702	-10.4354506615	-2.4532274218
O	23.0859961524	-10.1353193478	0.9468736144
N	17.4976783684	-8.6572152566	-1.7378622368
O	15.1179563972	-9.4067899143	-2.6236496488
O	15.6653583451	-11.604336017	-2.8176741815
H	8.7947728445	-5.6737800738	-0.7132610239
H	10.4175238193	-9.6396008785	-0.2677402469
H	12.6869651615	-8.6774191899	0.0498231043
H	11.0780303535	-4.7055362137	-0.3999342221
H	13.4180115546	-5.1534687752	0.6297918298
H	14.1068423788	-6.7751136895	0.4470862097
H	13.4290619885	-4.7737200767	-1.7780304721
H	8.1171550091	-9.1452193837	-0.6630140698
H	14.066436175	-6.4501768298	-3.2904547311
H	21.0101526144	-11.4962856207	1.6308065777
H	22.7267186095	-9.1626749436	-1.5560719861
H	20.6961484787	-9.5489024692	-2.9357634348
H	18.967618916	-11.8827284542	0.2458644497
H	18.5945328745	-10.6695730553	-3.3090183504
H	18.1205998931	-11.9974464648	-2.248279017

H	17.0737417715	-10.4073682485	-0.6633829559
H	23.7851203158	-9.5938736493	0.5321201313
H	18.0907153623	-8.3955678411	-2.532680654
H	13.2696631336	-7.3883626599	-2.1768922788
H	17.9502759323	-8.3236137027	-0.8835526763

Table S6. The calculated energies, sums of monomers, interaction energies, and Gibbs free energy of possible complexes of H(Tyr) and Tyr molecules with a copper(II) ion in an aqueous solution.

Interaction scheme of H(Tyr)– and Tyr–Cu(II) complex	Energy [Hartree]	Sum of monomers [Hartree]	Interaction energy [kcal/mol]	ΔG [Hartree]
Cu_H_(Tyr)$_2$_1	-1454.905932	-1454.469047	-274.1	-1454.609125
Cu_H_(Tyr)$_2$_2	-1454.880325	-1454.476833	-253.2	-1454.587386

Table S7. Atomic coordinates of optimized structures of complexes consisting of one H(Tyr) and Tyr molecule and a copper(II) ion from interaction schemes Cu_H_(Tyr)$_2$_1 and Cu_H_(Tyr)$_2$_2.

	Cu_H_(Tyr)$_2$_1			Cu_H_(Tyr)$_2$_2		
Atom	x	y	z	x	y	z
Cu	17.438050256	-8.2302602833	-1.7004256939	19.23126	-8.87684	-1.96496
C	12.6708734138	-7.1905564082	0.7398759499	11.60056	-6.63274	-0.75186
C	11.4533500696	-6.692036175	0.1542968677	11.36678	-7.99788	-0.92834
C	11.6365582096	-5.7251907233	-0.9008337897	12.44430	-8.87877	-1.04111
C	12.9019021617	-5.3151281911	-1.3224576783	13.75610	-8.39859	-0.97787
C	14.0807329202	-5.8230350487	-0.732776691	14.00790	-7.02283	-0.78774
C	13.9311895104	-6.7684314137	0.3049828154	12.91009	-6.14981	-0.68025
C	15.4409295469	-5.4148426305	-1.238771545	15.39602	-6.41087	-0.69801
C	15.8055553362	-6.1219735899	-2.571735057	16.60973	-7.36194	-0.83001
C	17.2515016097	-5.7672175496	-2.9269283907	17.91655	-6.58568	-0.83643
O	10.2656939415	-7.0832814899	0.5504037109	10.10741	-8.46287	-0.99262
N	15.6954032212	-7.5949680617	-2.3966630362	16.60284	-8.32572	0.28188
O	18.1604668578	-6.6463015929	-2.5333017729	19.08083	-7.20273	-1.12275
O	17.5181199011	-4.6775479129	-3.4876265275	17.93639	-5.39032	-0.58307
C	23.4543737418	-11.0642999678	-1.1644042976	24.89416	-13.49887	-0.53083
C	24.1427409692	-9.9908149384	-0.5780263125	25.66932	-12.35094	-0.72565
C	23.5070316868	-9.1650133204	0.360209844	25.04998	-11.15370	-1.10414
C	22.1713684573	-9.4200018693	0.7047196889	23.66680	-11.10537	-1.28276
C	21.4616361539	-10.4863340084	0.1288083422	22.87614	-12.25261	-1.08018
C	22.1242917854	-11.3029065247	-0.8094290809	23.50716	-13.45305	-0.70935

C	20.011466262	-10.7381991097	0.4674059367	21.37682	-12.21103	-1.28874
C	19.0793159763	-10.3133073739	-0.6777159398	20.62175	-11.34674	-0.25258
C	17.6055792563	-10.6642022455	-0.3954003073	19.13307	-11.38144	-0.54195
O	25.461423719	-9.8027399301	-0.9703479966	27.00489	-12.40985	-0.55264
N	19.1859520679	-8.8605325383	-1.0020766073	21.13251	-9.97736	-0.20338
O	16.7171730157	-9.7962852478	-0.8458613986	18.46089	-10.31971	-1.04026
O	17.3090425312	-11.7319352367	0.1896778475	18.51647	-12.41555	-0.33657
H	12.582576359	-7.9176263069	1.5467397444	10.76880	-5.94466	-0.66923
H	10.7407129044	-5.31483754	-1.3665520343	12.26677	-9.93796	-1.18040
H	12.9893250647	-4.5791484907	-2.1242874628	14.55032	-9.11650	-1.08200
H	14.8272646059	-7.1689946229	0.7864067784	13.07031	-5.08725	-0.54217
H	15.4966075382	-4.3356698384	-1.4319613435	15.46488	-5.65827	-1.51504
H	16.214880697	-5.6576259023	-0.4938195061	15.47407	-5.87698	0.27524
H	15.1366453581	-5.7619437146	-3.3620567	16.52871	-7.88281	-1.81092
H	14.9399243889	-7.8306249768	-1.7408263203	16.48537	-9.29888	-0.07438
H	23.9712313177	-11.6924401958	-1.8830635828	17.47990	-8.29730	0.85261
H	24.0441426715	-8.336375766	0.8158216008	25.36713	-14.42894	-0.24292
H	21.6810786815	-8.7826318897	1.4378132366	25.63836	-10.25914	-1.26595
H	21.5936535312	-12.1399890134	-1.2599774719	23.22686	-10.16970	-1.59285
H	19.8273941676	-11.8024153787	0.6545565575	22.92796	-14.35497	-0.55576
H	19.7249063644	-10.1976292905	1.3802489775	21.17723	-11.83992	-2.31797
H	19.3547543607	-10.8669440428	-1.5855492671	20.96362	-13.24238	-1.22360
H	25.8815437566	-9.0391679583	-0.5296658461	20.77620	-11.77912	0.76170
H	19.924239885	-8.6651261978	-1.6823556642	27.57329	-11.63690	-0.68170
H	15.5221860079	-8.0794639813	-3.2809877837	21.08859	-9.51857	-1.13650
H	19.3751097614	-8.3046215113	-0.1612927929	20.59779	-9.42101	0.50388

Table S8. Calculated energies, sums of monomers, interaction energies, and Gibbs free energy of possible complexes of Tyr and Ado molecules with a copper(II) ion in an aqueous solution.

Interaction scheme of Tyr– and Ado–Cu(II) complex	Energy [Hartree]	Sum of monomers [Hartree]	Interaction energy [kcal/mol]	ΔG [Hartree]
Cu(Tyr)(Ado)_1	-1788.628790	-1788.237692	-245.4	-1788.619076
Cu(Tyr)(Ado)_2	-1788.631529	-1788.230676	-251.5	-1788.629787

Table S9. Atomic coordinates of optimized structures of complexes consisting of Tyr and Ado molecules and a copper(II) ion from interaction schemes Cu(Tyr)(Ado)_1 and Cu(Tyr)(Ado)_2.

	Cu(Tyr)(Ado)_1			Cu(Tyr)(Ado)_2		
Atom	x	y	z	x	y	z
C	63.57390033	-15.6510457001	-3.5741094772	-6.9104548265	0.9233094032	-0.9382163197
C	62.6913090559	-16.2384786781	-2.5835970301	-7.089351512	1.9333988484	0.0871240185
C	62.1663962302	-15.3818564296	-1.5429231382	-6.5056123879	1.6811497318	1.3893974291
C	62.5597798921	-14.0586458337	-1.4651739189	-5.8023127221	0.5140973502	1.6326885965
C	63.4615730817	-13.5063894771	-2.4145908189	-5.6342102234	-0.4621962885	0.614828153
C	63.9340126966	-14.3229329831	-3.4851513316	-6.201908604	-0.2314078497	-0.6707140551
O	62.3921178871	-17.4967263927	-2.6146535210	-7.7476006433	3.0184550053	-0.1510171184
C	63.9739485142	-12.1049191825	-2.2433270388	-4.8389268287	-1.7121455374	0.8638589467
C	65.5019392446	-12.0847418486	-2.0690564710	-3.4672364971	-1.6628745552	0.1622554648
N	65.9612354032	-13.0720070634	-1.0312874710	-2.6069496276	-0.5498868834	0.6573718778
C	66.0192224465	-10.6660870297	-1.6941971988	-2.7339607412	-3.0254144328	0.2705765675
O	65.5765377366	-9.6979990726	-2.3956727578	-3.4243386516	-4.0822185848	0.2234052211
O	66.8710949151	-10.6201662526	-0.7308866795	-1.4385903014	-2.9670473934	0.3592809781
Cu	66.7257908913	-14.6294073333	-1.7907291124	-0.7241841514	-0.9283967596	0.2604185407
N	71.3485167189	-15.3199427808	-3.0470514818	3.3902281127	-3.6492785143	-0.4977689619
C	71.5773222621	-16.5086768933	-3.6516397833	4.4778796348	-2.8802927331	-0.7155351291
N	70.6774936680	-17.4809537861	-3.9093687940	4.5364635295	-1.5298993212	-0.7417840344
C	69.4376412278	-17.1514835031	-3.4877237879	3.3262529345	-0.9717657887	-0.5204553163
C	69.0641386409	-15.9593196445	-2.8519879140	2.1184346043	-1.6453454905	-0.2790157589
C	70.0884994142	-15.0024607437	-2.6304848531	2.1675305116	-3.0687672407	-0.2715324946
N	68.2766900169	-17.9015345191	-3.6073862601	3.0279380446	0.3838480693	-0.4727752981
C	67.2488146972	-17.1832922530	-3.0538396752	1.6863203027	0.501117235	-0.2077081458
N	67.6863481950	-16.0028713771	-2.5901337356	1.103164488	-0.6952976645	-0.0880195115
N	69.9027052609	-13.8011132720	-2.0393920604	1.1178536875	-3.880020292	-0.0597903804
C	66.8240238553	-20.6214455433	-2.7661690531	4.629795086	3.5672744464	0.3033286491
C	68.2907571771	-20.3381592713	-3.0771510789	4.4757763362	2.0927373806	0.6675224477
C	68.2281236067	-19.2571765376	-4.1698216005	3.9880060467	1.4673214353	-0.6596568993
O	66.9831831652	-19.4562347393	-4.8451229848	3.352142451	2.5295712576	-1.3964915363
C	66.1529879645	-20.4545051001	-4.1364507522	3.4679655199	3.8003507128	-0.653862754
O	69.0551316154	-19.8659208989	-1.9676037624	5.7370913403	1.5860565506	1.1060590056
O	66.7309837172	-21.9561842933	-2.2447353787	4.5011090714	4.459142025	1.413477116
C	64.7221454905	-19.9692351283	-4.1050492364	3.6889379779	4.9014593601	-1.6667147611
O	64.6568213456	-18.7981908565	-3.2720132831	2.5504713957	5.0381838745	-2.5431873366
H	63.9359678164	-16.2873519114	-4.3769980304	-7.3552299832	1.110784898	-1.9112753226

H	61.4882862213	-15.8242809535	-0.8198213516	-6.6423685752	2.4376252544	2.1567821757
H	62.1899513794	-13.4259507815	-0.6628965882	-5.3719732717	0.330110059	2.6140086733
H	64.5913461652	-13.8876308543	-4.2344107055	-6.0791118666	-0.9882856859	-1.4422952926
H	63.4999314603	-11.6280839531	-1.3764658143	-5.3618940121	-2.5928760778	0.4734394108
H	63.7468626394	-11.4866385477	-3.1198707905	-4.6841355047	-1.872303314	1.9388431772
H	65.9745640549	-12.3364820959	-3.0273889260	-3.6187094963	-1.4914171618	-0.9128963382
H	66.6560703036	-12.5433320579	-0.4683567736	-2.8938550956	0.3464610572	0.2579043889
H	65.1935875604	-13.3254857440	-0.3992029432	-2.6627209079	-0.4741653767	1.678488566
H	72.5993812270	-16.6967380195	-3.9621115912	5.410008752	-3.4082597709	-0.8873105594
H	66.2237069730	-17.5551833975	-3.0414869458	1.1832951812	1.4524243428	-0.1317918947
H	70.6978049431	-13.1841172664	-1.9284783444	1.301097727	-4.8767444885	-0.0799857628
H	68.9971219428	-13.4932061973	-1.7073601562	0.1506804809	-3.5594915699	0.1065824164
H	66.4354204029	-19.8809971847	-2.0530131993	5.5954623972	3.6922984964	-0.2138367301
H	68.7221966003	-21.2531229580	-3.5089351784	3.6976366103	1.989291885	1.4401068731
H	69.0620879072	-19.3234949206	-4.8731837930	4.8368106473	1.0521046388	-1.216792844
H	66.2110617658	-21.4053168154	-4.6819899603	2.5417201462	3.9721289645	-0.0865794851
H	69.2087797074	-20.5880790592	-1.3274226689	5.6511133081	0.7367378282	1.5811398661
H	65.8985364060	-22.0969909373	-1.7547886228	5.2841593828	4.4044672823	1.9954469242
H	64.3924935543	-19.7478472740	-5.1289394472	3.8009387093	5.8575343623	-1.148580557
H	64.0915483662	-20.7774911675	-3.7054266515	4.6070441366	4.6910876854	-2.2355152249
H	63.7310062386	-18.4593734848	-3.1492700768	2.4152268471	4.2057013551	-3.038090661

Table S10. Calculated energies, sums of monomers, interaction energies, and Gibbs free energy of possible complexes of H(Tyr) or Tyr molecules and AMP with copper(II) ion in aqueous solution.

Interaction scheme of H(Tyr) or Tyr and AMP – Cu(II) complex	Energy [Hartree]	Sum of monomers [Hartree]	Interaction Energy [kcal/mol]	ΔG [Hartree]
Cu(Tyr)H(AMP)$^{-}$_2	-2355.692209	-2355.236054	-286.2	-2355.316372
Cu(Tyr)(AMP)$^{2-}$_2	-2355.229275	-2354.756176	-296.9	-2354.862743

Table S11. Atomic coordinates of optimized structures of complexes consisting of H(Tyr) or Tyr and AMP molecules and copper(II) ion from interaction schemes Cu(Tyr)H(AMP)$^{-}$_2 and Cu(Tyr)(AMP)$^{2-}$_2.

	Cu(Tyr)H(AMP)$^{-}$_2				Cu(Tyr)(AMP)$^{2-}$_2		
Atom	x	y	z	Atom	x	y	z
N	24.0785939653	-13.7452507585	-1.0759412370	C	68.1021325978	-14.8246317612	-4.9105215186
C	24.4134400283	-15.0503389823	-1.1626451114	C	69.3457276661	-14.6480636705	-5.6091272949
N	23.7077857425	-16.0420959030	-1.7541802881	C	69.9518492954	-13.3502979375	-5.4579995344
C	22.5572008008	-15.6004870942	-2.3194503621	C	69.3872552218	-12.3570335631	-4.6574497651
C	22.1013046216	-14.2744536354	-2.2924157964	C	68.1814657394	-12.5666492128	-3.9455298493
C	22.8983043060	-13.3133137121	-1.6204679463	C	67.5500753473	-13.8168132361	-4.1129725464
N	21.5915920853	-16.3257978801	-3.0194769161	O	69.8941182948	-15.6031440035	-6.3303474437
C	20.6367457582	-15.4231820131	-3.4120343371	C	67.5501774216	-11.47547764	-3.1086461086
C	13.0463813804	-11.7672703476	-3.3308125937	C	68.1562484846	-11.2765350439	-1.694246378
C	12.5068377291	-12.0534912548	-2.0694577409	N	68.0552409707	-12.5283155221	-0.8915959638
C	13.2074922230	-11.7128808477	-0.9010241085	C	69.6278887808	-10.8727300017	-1.8281095376
C	14.4550035050	-11.0863884527	-1.0084906415	O	69.9307802911	-9.7094902279	-2.1879377806
C	15.0167987355	-10.7889679835	-2.2630966088	O	70.5050099327	-11.8303364058	-1.5907701632
C	14.2949523561	-11.1394863308	-3.4183791744	Cu	69.7037236233	-13.5875317798	-1.2664671572
O	11.2649604033	-12.6751714632	-2.0410152765	N	74.1039087898	-13.9537480249	-4.0858947902
C	16.3904313280	-10.1676005577	-2.3567286358	C	74.1565133355	-15.1582223808	-4.6901747334
C	17.4741434695	-11.1845730340	-1.9730472904	N	73.213986554	-16.1252004642	-4.6555279671
N	17.4458080818	-12.4091255901	-2.8105330153	C	72.1387041852	-15.7839887803	-3.9037036386
C	18.8991593214	-10.5953012471	-2.0168367977	C	71.9849133255	-14.5871051721	-3.1891316366
O	19.1180974904	-9.3899042720	-1.7675658626	C	73.0141397388	-13.6203436095	-3.3211924595
O	19.8518107809	-11.4770738523	-2.3036799556	N	70.9972426977	-16.5324814933	-3.6684438495
N	20.8829485374	-14.1902429442	-2.9692919729	C	70.183090224	-15.7758527994	-2.875462647
N	22.5414945076	-12.0221738878	-1.5000308397	N	70.7609949958	-14.6264887434	-2.5199000645
C	20.1654748328	-19.1287529042	-1.5959467178	N	72.9694327283	-12.4022880979	-2.7510727408
C	21.6081085644	-18.7036682827	-1.9924740415	C	71.1738943672	-19.2832460126	-2.5022143707
C	21.3846100391	-17.7890667451	-3.2618045107	C	71.5532209486	-18.9694753156	-3.95218756
O	19.9957875359	-17.9273068097	-3.5688742783	C	70.5753218205	-17.8206343159	-4.2764607215
C	19.3343478916	-18.0399719051	-2.2478913771	O	69.3533230589	-18.2209155298	-3.6411539133
O	22.1770090219	-18.0637136934	-0.8538509538	C	69.6424655259	-19.1677601022	-2.535574842
O	19.7756510330	-20.3923296283	-2.1608569642	O	72.9443895892	-18.7462800543	-4.1322991367
C	17.8459718248	-18.2228456327	-2.4366748971	O	71.6065015694	-20.6139935758	-2.1768101372
O	17.2182531457	-16.9755844543	-2.8365663504	C	68.9589715776	-18.744649352	-1.248341961
Cu	19.1762311554	-13.2948312316	-2.5015150230	O	69.5229621771	-17.5246045714	-0.7237199584
P	17.0806429169	-15.6610847781	-1.7021579842	P	68.425630835	-16.3126278424	-0.0835223174

O	18.5654217625	-14.9497367550	-1.7979787690	O	69.4219234444	-15.0121649257	-0.0306073174
O	16.8340324474	-16.2956635267	-0.2658999594	O	67.9911869456	-16.7880795222	1.3705174984
O	15.9616038038	-14.6957112919	-2.3181498329	O	67.2793451978	-16.1388190683	-1.1843532089
H	25.3501521841	-15.3376916125	-0.6978155613	H	67.5938798478	-15.7838281935	-5.0124953006
H	19.7601268118	-15.7244466912	-3.9587707561	H	70.8861340172	-13.1643629268	-5.9903326832
H	23.1614214085	-11.3953854165	-1.0015104104	H	69.8865067652	-11.3884716532	-4.5786849612
H	21.6338351793	-11.6767410200	-1.8410913334	H	66.5979390152	-14.000044256	-3.6113498596
H	20.0859542986	-19.1225462899	-0.5029097511	H	67.6316031631	-10.5009692282	-3.6089626493
H	22.1945042236	-19.5755998060	-2.3097154805	H	66.4781364568	-11.678495305	-2.9747454401
H	21.9706214945	-18.1050613224	-4.1242792021	H	67.6228127976	-10.4528811388	-1.2057708617
H	19.5029493017	-17.1029010033	-1.6905958625	H	67.2526182778	-13.1052840358	-1.1610829631
H	23.0264328711	-17.5911939736	-1.0667244013	H	68.0053606067	-12.3467629703	0.1130756882
H	20.2142804338	-21.1330469272	-1.7003679457	H	75.051400642	-15.3717706864	-5.2643892118
H	17.6441054107	-18.9389507235	-3.2371025983	H	69.1936052914	-16.096598668	-2.5927101956
H	17.4060649582	-18.5907006605	-1.4997986997	H	73.7513301587	-11.7765754013	-2.9016653116
H	12.4853999636	-12.0390158060	-4.2194785357	H	72.1272447314	-12.0709012012	-2.2559915184
H	12.7838787659	-11.9335538079	0.0760733718	H	71.6159100488	-18.5373779154	-1.825829946
H	14.9951177657	-10.8192441616	-0.1017647174	H	71.2387225893	-19.8271632822	-4.5677218014
H	14.7133101450	-10.9180051279	-4.3982179860	H	70.3850334502	-17.6203195553	-5.3366774295
H	10.9513442792	-12.8509729502	-1.1329055836	H	69.2379960567	-20.1458151666	-2.8315956946
H	16.4880267333	-9.3075900021	-1.6837565067	H	73.1679760319	-17.852341328	-4.5270696016
H	16.5848476969	-9.8050775391	-3.3755087273	H	71.6710947736	-20.7410005575	-1.2114237235
H	17.3074095706	-11.5089186894	-0.9345641682	H	69.0914141608	-19.5570841114	-0.5190698598
H	17.3804782723	-12.1733285375	-3.8060548964	H	67.8831238186	-18.6201326589	-1.4404531609
H	16.6985050919	-13.1053882486	-2.5733960798				

Table S12. Calculated energies, sums of monomers, interaction energies, and Gibbs free energy of possible complexes of Tyr and ADP molecules with copper(II) ion in aqueous solution.

Interaction scheme of Tyr and ADP – Cu(II) complex	Energy [Hartree]	Sum of monomers [Hartree]	Interaction Energy [kcal/mol]	ΔG [Hartree]
$Cu(Tyr)(ADP)^{3-}$_2	-2922.249051	-2921.754231	-310.5	-2921.906893
$Cu(Tyr)(ADP)^{3-}$_3	-2922.237163	-2921.770783	-292.7	-2921.869332

Table S13. Atomic coordinates of optimized structures of complexes consisting of Tyr and ADP molecules with copper(II) ion from interaction schemes Cu(Tyr)(ADP)$^{3-}$_2 and Cu(Tyr)(ADP)$^{3-}$_3.

	Cu(Tyr)(ADP)$^{3-}$_2			Cu(Tyr)(ADP)$^{3-}$_3		
Atom	x	y	z	x	y	z
C	-8.18886	-0.69270	-0.02653	15.8391861054	-23.3803105368	-3.5490067300
C	-8.63713	-0.22828	1.25965	15.9602116425	-21.9506924239	-3.4203843240
C	-7.67160	0.55217	1.98984	17.0003495397	-21.5106600926	-2.5276471878
C	-6.40085	0.83116	1.47829	17.8308992246	-22.4095453895	-1.8487935641
C	-5.98411	0.35937	0.21446	17.6951439121	-23.8035336351	-1.9968427519
C	-6.91064	-0.40526	-0.52053	16.6759186846	-24.2625411676	-2.8597611816
O	-9.83711	-0.49088	1.73195	15.1773838083	-21.1141961002	-4.0662871015
C	-4.61507	0.71100	-0.32196	18.5637953713	-24.7841368140	-1.2401869672
C	-3.43371	0.12783	0.49288	19.4369596468	-25.6739539168	-2.1462757151
N	-2.22809	0.99080	0.42949	19.7861573519	-26.9834199362	-1.5243537822
C	-3.05849	-1.30035	0.06413	20.7585197806	-25.0187825303	-2.5832005550
O	-3.93438	-2.13713	-0.26423	20.9055340412	-23.7814508967	-2.6665358354
O	-1.76759	-1.58517	0.10398	21.7245869060	-25.8856360368	-2.8776850086
Cu	-0.54560	-0.05911	0.28830	21.3000423362	-27.7549295797	-2.5908178162
N	2.09239	-4.09951	-1.45274	26.4607498563	-26.9050772849	-2.9166490318
C	3.39766	-3.97425	-1.14579	27.1057941236	-28.0710877113	-3.1417748901
N	3.95719	-3.07188	-0.30851	26.5547672538	-29.2776168952	-3.3987067499
C	3.05028	-2.21988	0.23515	25.2042876418	-29.2277427138	-3.3944664959
C	1.67227	-2.22313	-0.03404	24.4054249830	-28.0941522112	-3.1519377660
C	1.17978	-3.23186	-0.90552	25.0907246000	-26.8689553232	-2.9256824848
N	3.26251	-1.19325	1.15013	24.3230093101	-30.2730869819	-3.6235579534
C	2.03336	-0.63109	1.39796	23.0587721529	-29.7794780803	-3.4944344868
N	1.05874	-1.19725	0.69424	23.0537958631	-28.4754641234	-3.2027967997
N	-0.12030	-3.38474	-1.19993	24.4731295227	-25.6902197236	-2.7298322392
C	6.24193	0.82904	0.92732	24.0492810043	-33.5009056250	-2.1941539704
C	5.79480	-0.65548	0.90974	25.0416576254	-32.3197380393	-2.3485561506
C	4.47917	-0.62396	1.76866	24.6335152124	-31.7013291455	-3.7291931148
O	4.20657	0.77205	2.02227	23.4344570808	-32.3827536064	-4.1274094939
C	4.91286	1.58618	1.02330	22.8191376963	-32.9437582104	-2.9002683688
O	5.64535	-1.03986	-0.45387	24.8169531729	-31.3345997964	-1.3300590882
O	7.09180	1.11374	2.05427	24.5437813560	-34.7024744599	-2.8177301733
C	4.19745	1.68651	-0.34888	21.7234623402	-33.8955090692	-3.3112264217
O	2.80504	1.31690	-0.29162	20.6049625231	-33.1378581971	-3.8326844563
P	1.53010	2.41710	-0.05160	19.2181574314	-32.8419959023	-2.8570539323

O	0.44313	1.61027	0.82122	18.2011155689	-32.0671995489	-3.7834092415
O	0.92503	2.62646	-1.58732	19.8928099351	-31.8204442155	-1.6869310983
O	2.08590	3.77009	0.53672	18.8120958699	-34.1829328028	-2.1219335396
P	-0.33421	1.67157	-2.44129	19.9400537166	-30.1512441296	-1.3058812366
O	-1.71856	2.32968	-1.95661	20.5100798464	-29.4916376772	-2.7056987906
O	0.04756	1.85401	-3.96972	21.0030733742	-30.0345558896	-0.1268691589
O	-0.11655	0.19251	-1.81800	18.4624392348	-29.6181651947	-1.0154597689
H	-8.88582	-1.28652	-0.61875	15.0595580901	-23.7626133094	-4.2084790791
H	-7.96716	0.93361	2.96772	17.1287272600	-20.4362048999	-2.3926316979
H	-5.71254	1.44177	2.06804	18.6088830687	-22.0253837889	-1.1876029068
H	-6.61685	-0.78395	-1.50050	16.5295880171	-25.3384425375	-2.9831848447
H	-4.52000	0.39399	-1.36973	17.9307814837	-25.4682440288	-0.6530416900
H	-4.48282	1.80450	-0.30670	19.2102269291	-24.2515469496	-0.5295259392
H	-3.74486	0.05326	1.54438	18.8780261170	-25.9037830156	-3.0643130649
H	-2.14245	1.60756	1.23837	19.0011112422	-27.6574072445	-1.4838467738
H	-2.17888	1.56324	-0.45098	20.1338518342	-26.8524509020	-0.5676740929
H	4.07585	-4.67305	-1.62352	28.1898837900	-28.0290129326	-3.1170389343
H	1.90636	0.21229	2.05270	22.1564517871	-30.3609671506	-3.5998131917
H	-0.38356	-4.13348	-1.82894	23.4462347139	-25.6074631092	-2.7856366928
H	-0.84138	-2.77340	-0.78051	25.0482581674	-24.8711521246	-2.5766042068
H	6.82247	1.05593	0.03263	23.8804392448	-33.7428706604	-1.1443212511
H	6.53911	-1.27347	1.42651	26.0765343035	-32.6768694135	-2.3636910677
H	4.63260	-1.10105	2.73953	25.4013845337	-31.8446990189	-4.4907912502
H	5.02226	2.56835	1.48874	22.3846129579	-32.1364143802	-2.2898136984
H	5.29817	-1.97581	-0.52275	25.2637247252	-31.5779909048	-0.4969769984
H	6.57913	1.10588	2.88862	24.5902130666	-34.6007263655	-3.7909864642
H	4.64449	0.98278	-1.05534	22.0650256577	-34.5576675950	-4.1129709162
H	4.31858	2.70897	-0.72707	21.4183063632	-34.5091180534	-2.4516198162

Table S14. Calculated energies, sums of monomers, interaction energies, and Gibbs free energy of possible complexes of H(Tyr) and ATP molecules with copper(II) ion in aqueous solution.

Interaction scheme of H(Tyr) and ATP – Cu(II) complex	Energy [Hartree]	Sum of monomers [Hartree]	Interaction Energy [kcal/mol]	ΔG [Hartree]
Cu(Tyr)H(ATP)$^{3-}$_1	-3489.7597	-3489.284827	-298.0	-3489.369610
Cu(Tyr)H(ATP)$^{3-}$_2	-3489.759254	-3489.281271	-299.9	-3489.370832

Table S15. Atomic coordinates of optimized structures of complexes consisting of H(Tyr) and ATP molecules and copper(II) ion from interaction schemes $Cu(Tyr)H(ATP)^{3-}$_1 and $Cu(Tyr)H(ATP)^{3-}$_2.

	$Cu(Tyr)H(ATP)^{3-}$_1			$Cu(Tyr)H(ATP)^{3-}$_2		
Atom	x	y	z	x	y	z
C	23.0153208638	-18.2660211947	-0.1904030114	23.5240160528	-18.3321838754	-1.1679588549
C	22.3396490468	-19.0301135297	-1.1610646383	22.9473622915	-18.0939834299	-2.4232347048
C	22.900071091	-19.1439894859	-2.4527042357	22.5331090294	-19.1643693374	-3.230534349
C	24.133078024	-18.5499058732	-2.7378969781	22.7070287908	-20.4757894258	-2.769523104
C	24.8425725242	-17.8250080466	-1.759924125	23.2915692595	-20.7399732426	-1.5178128844
C	24.2546997654	-17.6855923083	-0.4897045071	23.6929837212	-19.6494133461	-0.726495425
O	21.1697245089	-19.6516035834	-0.8302337789	22.8087968595	-16.7667123176	-2.813688701
C	26.2047643313	-17.227027556	-2.038624366	23.4557751061	-22.1673120591	-1.0459400366
C	27.1248834774	-18.1744111675	-2.8213185989	24.4500220439	-22.9782099114	-1.8951229533
N	27.1687362621	-19.5567847165	-2.2706476114	24.2359513254	-24.4454981136	-1.8114399849
C	28.5711804611	-17.6537917122	-2.8945455721	25.9228025023	-22.6970324903	-1.5590765474
O	28.8322578956	-16.4339079197	-2.9264679183	26.3097825425	-21.6088031414	-1.0813046347
O	29.5012932996	-18.6062188847	-2.9429997235	26.7383181773	-23.7043386206	-1.8454328925
Cu	28.7812168673	-20.4011439833	-3.0372179633	25.869540569	-25.3039342799	-2.55871519
N	33.25634452	-20.6375072501	-0.5884242345	31.1645999374	-25.422854418	-2.4879763683
C	33.6402294095	-21.9262722115	-0.4626718684	31.59554585	-26.6889745973	-2.650143646
N	33.092912017	-23.0075101632	-1.0602900403	30.831905037	-27.797463411	-2.7849457575
C	32.0444636478	-22.6775688168	-1.8481719899	29.5070559546	-27.5030598286	-2.7694177151
C	31.5295849811	-21.3858611225	-2.0528483565	28.9204998301	-26.2363236886	-2.6186851537
C	32.1917686261	-20.3172558191	-1.388343184	29.8201563684	-25.1464394462	-2.4447918738
N	31.2579594517	-23.5317542029	-2.6171665979	28.4602233398	-28.3998612528	-2.9007041023
C	30.3361624274	-22.7614134168	-3.2607664773	27.2941120986	-27.6908837062	-2.8498532592
N	30.4355489893	-21.4682344047	-2.9310219713	27.5240796763	-26.3843270829	-2.6757327274
N	31.8437285435	-19.0235859586	-1.5085787671	29.4345433849	-23.8775744426	-2.2380718855
C	29.5646791126	-25.4203888827	-0.8919851475	28.8551220741	-31.7357908061	-1.6388606272
C	31.0791222463	-25.5078259074	-1.1160114746	29.5735540017	-30.4441447437	-2.0320326051
C	31.2276744204	-25.0148875596	-2.5738880758	28.6120712922	-29.848043252	-3.0873795457
O	30.0494639538	-25.4619083316	-3.2487756446	27.3407756005	-30.4743821651	-2.8952123528
C	29.010683248	-25.8556865849	-2.2620679333	27.4022913977	-31.2588755239	-1.6509842292
O	31.428914398	-26.8986697254	-1.048704642	30.8895253915	-30.6662117042	-2.5260033018
O	29.2249860325	-24.0787593189	-0.4940629102	29.1809360329	-32.2034337755	-0.3271212091
C	27.6581874164	-25.3130555926	-2.6824690748	26.3252275628	-32.312017589	-1.6759565998
O	27.7110192788	-23.8560936686	-2.6324119285	25.0817751543	-31.7106789532	-1.246986237
P	26.7594306167	-22.8547798227	-3.623664796	23.6966636144	-31.5892149413	-2.2541041812

O	27.7937951738	-21.7056015816	-4.0630752806	24.1320800959	-31.4811245242	-3.7701887643
O	25.7436162279	-22.10024044	-2.5411103127	23.1168107186	-30.119155564	-1.6469556064
O	26.0410197769	-23.6709809019	-4.7540354155	22.6458630352	-32.684783824	-1.8157682086
P	24.5564815395	-22.8505374469	-1.466685917	23.4206330479	-28.4540928449	-1.6058174896
O	24.608240981	-21.9644928619	-0.1616694342	24.919370961	-28.1531619596	-1.1768735587
O	23.2266872715	-22.5701547014	-2.4236520754	23.2368903526	-28.1308290381	-3.255760949
O	24.9454409273	-24.386607818	-1.4234395027	22.2433525594	-27.8136975085	-0.7691355812
P	21.5184490928	-23.0234434713	-2.1577891624	23.4764782755	-26.7033229336	-4.2095195422
O	20.7640508411	-21.6019179483	-2.2946503712	25.0541720643	-26.3449876642	-3.9336633887
O	21.5242148482	-23.6253391062	-0.6772656892	22.5175691296	-25.5708738074	-3.5975438451
O	21.1906884187	-24.0182193209	-3.3560276879	23.2131540327	-27.1604158725	-5.7011886265
H	22.576723316	-18.1721411062	0.7993270595	23.8344320238	-17.4873802873	-0.56084831
H	22.3806598671	-19.7398102314	-3.1985907528	22.0777113075	-18.9758228553	-4.2002944102
H	24.544904102	-18.6616745282	-3.7406501011	22.3712612888	-21.3042216661	-3.3914998839
H	24.7830422152	-17.1275219797	0.2815204972	24.1508294011	-19.8357104932	0.2415144823
H	20.9588881683	-20.4732159207	-1.4649635164	22.4085204524	-16.6765273235	-3.6999170691
H	26.1286217595	-16.2968074482	-2.6159898912	23.7723706331	-22.1906683984	0.0043374165
H	26.6904317426	-16.9616856253	-1.0891752338	22.4882165094	-22.6856441256	-1.1099490013
H	26.7746893363	-18.261377626	-3.8591022278	24.3159121203	-22.7104748116	-2.9545531004
H	27.2654972297	-19.5444827112	-1.2490682977	24.1267845201	-24.7543303659	-0.8403532005
H	34.4871676195	-22.111129351	0.1897655909	32.6701552486	-26.8338237914	-2.6690733856
H	29.5954055271	-23.1728727849	-3.9212682187	26.3106623724	-28.1330054494	-2.8991635392
H	31.0466883599	-18.7237601716	-2.0856237137	28.439066841	-23.6427704748	-2.0903153939
H	32.3828155885	-18.337732435	-0.9942803305	30.1544994173	-23.1742924237	-2.1256319723
H	29.261540122	-26.1264270785	-0.1134507019	29.0273362864	-32.5046427534	-2.4085402786
H	31.6379025841	-24.8937443646	-0.4039934348	29.5834632451	-29.7903523403	-1.1434074073
H	32.113863022	-25.4178162324	-3.0702489717	28.9728740307	-30.0466567513	-4.1034206129
H	28.9555819935	-26.9498666046	-2.2780784198	27.2385387754	-30.5987185754	-0.7836202393
H	32.3939869074	-27.0270877828	-0.9767711309	31.2847864689	-29.7705115716	-2.6974365969
H	28.5629251526	-23.6900698384	-1.1177018308	30.0868404654	-32.5681144044	-0.3021792142
H	27.4226687218	-25.6344443513	-3.7031330699	26.242624789	-32.7391965883	-2.6849383633
H	26.865592473	-25.6480133725	-2.0029634648	26.5792148262	-33.1031473734	-0.9619347753
H	26.3484073377	-20.1361441349	-2.502480848	23.4227028339	-24.770132715	-2.3936159442

Table S16. Calculated energies, sums of monomers, interaction energies, and Gibbs free energy of possible complexes of Tyr and ATP molecules with copper(II) ion in aqueous solution.

Interaction scheme of Tyr and ATP – Cu(II) complex	Energy [Hartree]	Sum of monomers [Hartree]	Interaction Energy [kcal/mol]	ΔG [Hartree]
$Cu(Tyr)(ATP)^{4-}$_1	-3489.259014	-3488.816130	-277.9	-3488.886209
$Cu(Tyr)(ATP)^{4-}$_2	-3489.285936	-3488.801440	-304.0	-3488.905738
$Cu(Tyr)(ATP)^{4-}$_3	-3489.241025	-3488.833642	-255.6	-3488.868662
$Cu(Tyr)(ATP)^{4-}$_4	-3489.243233	-3488.822113	-264.3	-3488.871115

Table S17. Atomic coordinates of optimized structures of complexes consisting of Tyr and ATP molecules and copper(II) ion from interaction schemes $Cu(Tyr)(ATP)^{4-}$_1 and $Cu(Tyr)(ATP)^{4-}$_2.

	$Cu(Tyr)(ATP)^{4-}$_1			$Cu(Tyr)(ATP)^{4-}$_2		
Atom	x	y	z	x	y	z
C	14.2763883435	-18.3860328761	-2.3349406794	2.8040421656	0.30394479	2.065473161
C	13.144693496	-17.8819257477	-1.5855636083	2.4389379238	1.5570480197	2.6695329649
C	13.3216925021	-16.6336964506	-0.8667120134	3.0086442637	2.7197353223	2.0406835282
C	14.5292598565	-15.9665363714	-0.9032614359	3.8107403208	2.6325525496	0.9029685014
C	15.6304915818	-16.472155935	-1.654659628	4.110019863	1.3929421436	0.2908034552
C	15.474342636	-17.6923234217	-2.3683304931	3.5969266182	0.2351763367	0.9154430242
O	12.0197280856	-18.5177727036	-1.5509786428	1.651660907	1.6403590915	3.7195994877
C	16.9139351784	-15.6805699704	-1.6797082568	4.9757321231	1.3176770886	-0.9474437502
C	18.1252088012	-16.4377368896	-2.2335568386	4.2412917716	1.615627956	-2.2831341136
N	18.5083934181	-17.6067314031	-1.4005730159	3.1703966327	0.6237674481	-2.5189820193
C	19.3384960648	-15.4966943745	-2.3896184509	3.5839018402	2.9913011751	-2.2159896225
O	20.4611117256	-15.8814883514	-1.8812485628	4.2476760701	4.0450573885	-2.3732287029
O	19.1378804218	-14.4065354941	-3.0253670372	2.2903057658	2.9695017995	-1.9300431623
Cu	19.2605214287	-19.0443374481	-2.5433493473	1.6368059412	1.1924358379	-1.3722247203
N	24.0645200296	-19.4360793426	-1.2966967259	-0.040134989	5.0791491161	1.6721216103
C	24.5549319983	-20.6628287692	-1.5829513509	-1.0939873558	4.6714014067	2.4078292965
N	23.9482171673	-21.650949575	-2.2785848407	-1.7958160308	3.5288260772	2.2575259897
C	22.7109348988	-21.285802805	-2.6906388197	-1.2758616635	2.7400707178	1.2908297189
C	22.0767599566	-20.0566775827	-2.452531133	-0.146624752	2.9953632983	0.4973922949
C	22.8092862416	-19.0814720912	-1.7248146826	0.4458124486	4.2839017953	0.6642884287
N	21.8027451582	-22.0475770801	-3.4146533079	-1.7720376309	1.5064417382	0.8824059546

C	20.6660550317	-21.2825233402	-3.5956167562	-0.9411575041	1.0298144432	-0.1016408692
N	20.800424453	-20.0839982669	-3.0169574167	0.0590320014	1.8888467522	-0.3510421338
N	22.3687630759	-17.8436402045	-1.4293288019	1.4035070271	4.7955625389	-0.1238460304
C	20.2651921598	-24.6052990821	-2.5643870002	-4.1114891997	-0.5354111166	-0.170652648
C	21.7821231757	-24.3166873434	-2.4912371104	-4.142715281	0.9016172643	0.389981819
C	22.0201001557	-23.4616670252	-3.7396019879	-3.0018096302	0.9232387622	1.4183605491
O	21.0655248065	-23.9122307002	-4.7127880342	-2.7857615845	-0.4400754684	1.8194847108
C	20.0497311929	-24.7886585093	-4.0797669217	-3.6430771908	-1.3546474582	1.0494671601
O	22.5099691553	-25.565416393	-2.5342779123	-5.4139659534	1.0119156917	1.0656162783
O	19.920990672	-25.7415103613	-1.7688083391	-5.3974184972	-0.9143419926	-0.6707175627
C	18.6872519406	-24.3780196137	-4.6143539214	-2.7767248479	-2.5548835389	0.7200470058
O	17.7034940715	-25.1319604382	-3.8664313636	-3.5714090628	-3.403969305	-0.1365145241
P	16.3914635797	-24.3872941524	-3.0683796831	-2.8667688748	-4.7102931529	-0.985128859
O	15.2969345937	-23.9417982109	-4.1221005091	-2.346111747	-5.8073840161	0.0264527842
O	17.2240190258	-23.028384592	-2.470414915	-1.4826042929	-3.9558299603	-1.6190235205
O	15.9951236389	-25.3509203412	-1.8805857586	-3.8824503796	-5.0739602766	-2.1378622091
P	16.7452121929	-21.4579276627	-1.9894860654	-1.1129474642	-2.6666115228	-2.6723581496
O	18.0731657693	-20.7615926799	-1.4513851431	-1.7818508067	-1.3443020439	-2.0963522475
O	16.2649213765	-20.7513400537	-3.4228571727	0.5536996946	-2.6769250794	-2.4581682304
O	15.4880047641	-21.6011828731	-1.039895652	-1.4134214523	-3.0956234844	-4.1618423381
P	17.1723143559	-20.1450599872	-4.8131699258	1.6238626117	-2.0584622001	-1.2371419121
O	17.9308504158	-18.84535993	-4.2140202688	1.0136612646	-0.5623970493	-1.0200305779
O	18.1661571654	-21.3398745992	-5.2154448151	3.052460491	-2.0502596717	-1.9607735283
O	16.0210229572	-19.8000965578	-5.8571054883	1.4884327876	-2.9639337863	0.0588850319
H	14.1806836594	-19.3336574376	-2.8606649743	2.4144874451	-0.6099908426	2.5144159088
H	12.4754124153	-16.2553721613	-0.2997759135	2.7799736074	3.693277421	2.476425807
H	14.6565041931	-15.0361399052	-0.3544052951	4.2151822911	3.5496420563	0.4686100599
H	16.3011125438	-18.1014335927	-2.9518291625	3.8317142882	-0.742525472	0.4877651814
H	16.7723317163	-14.7827179367	-2.2995161479	5.806019283	2.0349073563	-0.8878026741
H	17.1329417462	-15.3143078187	-0.6643064683	5.4171595029	0.3153672072	-1.0437493495
H	17.8864055775	-16.8091572762	-3.2404824596	4.9808879118	1.6201014301	-3.0934568727
H	17.7107408115	-18.0426249221	-0.9309761836	3.4041969258	-0.371081777	-2.2925730655
H	19.2132501199	-17.3160371019	-0.7174731213	2.8302565162	0.6639755403	-3.4835702769
H	25.5525571674	-20.8753757183	-1.2121740854	-1.4200828824	5.3439832107	3.1946212003
H	19.7801198904	-21.5740286134	-4.1697691417	-1.0564426282	0.0718601833	-0.6101085969
H	22.9998586281	-17.2448537862	-0.9089477243	1.7499743596	5.721597666	0.0931988228
H	21.4724855203	-17.42921013	-1.7261550409	1.8127030068	4.2364150012	-0.8872610782
H	19.6738597319	-23.7590688251	-2.194518423	-3.3856947236	-0.6443067671	-0.987098094
H	22.0890952975	-23.8159614354	-1.5730639735	-4.0237629065	1.6767656634	-0.3733383228
H	23.0380563198	-23.5695353173	-4.1292269153	-3.2669488731	1.5385325974	2.2856471271
H	20.2683097253	-25.8308163824	-4.3555323704	-4.5113706805	-1.6433283686	1.6580749463

H	22.6403944165	-25.9004177621	-3.4449145995	-5.6870226885	1.9383558936	1.1996387379
H	20.6658803055	-26.3820235347	-1.8032765319	-6.0749801286	-0.4933992365	-0.0950219854
H	18.6021990028	-24.6482204745	-5.6728455339	-2.4840797906	-3.0876681907	1.6341501962
H	18.5290524955	-23.2928722668	-4.5208061	-1.8767471787	-2.206874998	0.1909690046

Table S18. Atomic coordinates of optimized structures of complexes consisting of Tyr and ATP molecules and copper(II) ion from interaction schemes Cu(Tyr)(ATP)$^{4-}$_3 and Cu(Tyr)(ATP)$^{4-}$_4.

	Cu(Tyr)(ATP)$^{4-}$_3			Cu(Tyr)(ATP)$^{4-}$_4		
Atom	x	y	z	x	y	z
C	16.1378202278	-15.0842365688	-3.2499333611	15.4639527777	-14.956687317	-1.0383667
C	16.9638670975	-15.1269431567	-4.4399784885	14.4432128338	-15.9716621357	-0.8610046021
C	18.2708897695	-15.7395745639	-4.3229862108	14.6998417028	-17.2868745124	-1.4147843526
C	18.7097236261	-16.2576381149	-3.1168840533	15.8759728906	-17.5590681362	-2.0888208296
C	17.8875189061	-16.2059124291	-1.9581524316	16.8625723311	-16.542453824	-2.2602340429
C	16.596110532	-15.6122264229	-2.0605142803	16.6323620646	-15.2471794683	-1.718221617
O	16.5515605242	-14.6389600339	-5.5621684233	13.3453139842	-15.7109884242	-0.2310135721
C	18.3178332866	-16.7125226171	-0.6035338925	18.1478359606	-16.860675155	-2.955658321
C	19.4984787306	-17.6881731406	-0.5715322056	19.1935960268	-17.4661946167	-1.9732477582
N	19.2365524529	-18.9483105641	-1.327481923	18.7096787968	-18.650225	-1.2493386115
C	20.8337492636	-17.0155897608	-0.9862749741	20.4419167323	-17.9441360196	-2.7291568059
O	21.0547699659	-15.8467406165	-0.5561812603	21.5978389695	-17.7873219549	-2.2228037924
O	21.641634432	-17.721491428	-1.7181222766	20.2026444765	-18.5898207308	-3.844476443
Cu	20.9066650193	-19.7556346212	-1.953969523	20.6808888859	-20.4392205598	-3.7496522618
N	25.5965955763	-20.0029247766	-3.684949577	25.0673985061	-21.565397996	-1.4863549318
C	25.9364060094	-21.2622957176	-4.036876901	25.1834528809	-22.8973378822	-1.276928551
N	25.175738486	-22.3750298524	-3.9418495272	24.2769620417	-23.860249338	-1.5555236501
C	23.9507683437	-22.108221456	-3.4372380527	23.1705588126	-23.3615322572	-2.1573860828
C	23.466742546	-20.8537508567	-3.0387488122	22.9554084037	-22.0081766306	-2.4833917909
C	24.3516499823	-19.7480022968	-3.1684973875	23.9317155705	-21.0681470096	-2.0689967155
N	22.9208425701	-23.0146858798	-3.2052155023	22.0316203952	-24.0378857321	-2.5904494516
C	21.8547517986	-22.307165564	-2.691869788	21.2115096169	-23.1084199722	-3.1860214994
N	22.1511725238	-21.0061617675	-2.581351622	21.7300820612	-21.8828184556	-3.1363221428
N	24.0577404348	-18.4815829661	-2.8220361741	23.8060088434	-19.7292390573	-2.1953933065
C	21.1936518461	-25.5475810392	-2.3106398585	20.618190059	-26.2474845588	-0.4028164414
C	22.7306417938	-25.3282234907	-2.280919621	22.0067695059	-26.0848115651	-1.0964032001
C	22.997963052	-24.449867616	-3.5085104474	21.6426859392	-25.4663253032	-2.4780294962
O	22.0157755711	-24.8379327803	-4.4769721665	20.2170439627	-25.5000671207	-2.572741552

C	20.7790686623	-25.2027713513	-3.7712698452	19.7284051068	-25.2983756817	-1.1970083362
O	23.4094780645	-26.5735798447	-2.4903500338	22.6540051579	-27.3491984943	-1.2481628678
O	20.9609732775	-26.9186408392	-1.9406149006	20.1993622984	-27.6248888058	-0.5578005394
C	20.1450675655	-26.3653405996	-4.5237177292	18.2366103909	-25.5343113206	-1.1422408867
O	19.0207729757	-26.8571213924	-3.742040503	17.9156956671	-26.8191418226	-1.7453632029
P	17.4394045711	-26.1839500534	-3.9764373226	16.9739110775	-26.9755687566	-3.1875091849
O	17.218846193	-25.9486724595	-5.5229892384	17.8317056991	-27.825409887	-4.2045782408
O	17.6738504246	-24.6644783166	-3.2813693708	16.9358991452	-25.3645929426	-3.7067814924
O	16.4718310273	-27.0923868838	-3.1270438336	15.5189966415	-27.4556132058	-2.7910421284
P	17.9368789126	-23.958757529	-1.7390750325	15.7544993402	-24.1080076499	-3.6881159097
O	19.146776551	-22.9428635215	-1.9575132441	15.1980416525	-23.9390575664	-2.2144594331
O	16.5607345796	-23.0805081196	-1.5162729294	16.7591695568	-22.8397547198	-4.1180430637
O	18.1488646565	-25.1194111803	-0.6809490698	14.7476394546	-24.3689095583	-4.8785153304
P	14.7649107481	-23.3571465913	-1.6073286573	17.8498392712	-21.7694289187	-3.2595500955
O	14.4507633743	-23.385934007	-3.176855217	18.9484960255	-21.4783985311	-4.4156906795
O	14.2478683211	-22.0553293201	-0.8346204416	18.4223712277	-22.6076464872	-2.0211789988
O	14.5430119641	-24.7462651028	-0.8423992181	16.9779517434	-20.4802253265	-2.8611704615
H	15.1550422113	-14.6294209246	-3.3335437316	15.2709602855	-13.9721069869	-0.6211986483
H	18.8908842499	-15.7743156293	-5.214340881	13.9329228349	-18.0442596434	-1.2748523357
H	19.7007390149	-16.7032221227	-3.0686225526	16.0749806204	-18.5535974658	-2.4946819813
H	15.9686137627	-15.5801477073	-1.1732919397	17.3944529258	-14.4826615371	-1.8512771558
H	18.5945890376	-15.8466911142	0.0143548961	17.9885024476	-17.6082989575	-3.7416109432
H	17.4539876868	-17.1777342769	-0.1101755077	18.5793594761	-15.9621623347	-3.4152768245
H	19.6510964447	-17.9751680418	0.4758552001	19.5025104707	-16.7098702386	-1.2416454761
H	18.7169419868	-19.6163513615	-0.7540741919	18.2411254602	-18.4292045207	-0.3742831313
H	18.6818418426	-18.7691529645	-2.1708840902	18.1578709031	-19.3012826362	-1.8314206747
H	26.9356070655	-21.3920608738	-4.4395809903	26.1112421476	-23.2271302167	-0.8207316731
H	20.8745050744	-22.7220460552	-2.4190429073	20.2409073502	-23.341748123	-3.5937929758
H	24.7807528874	-17.7868226987	-2.968253026	24.5723142897	-19.1600758993	-1.8547680442
H	23.1615434597	-18.1825143806	-2.4083281891	22.9372742728	-19.2449776388	-2.4572480335
H	20.6765879165	-24.8764434277	-1.6141522846	20.655759368	-26.0011737032	0.660075695
H	23.0717100889	-24.8531081808	-1.3551513608	22.6688490439	-25.4246055538	-0.5318324655
H	23.9932120028	-24.6293540523	-3.922531782	22.0718430514	-26.0430927178	-3.3002143541
H	20.103565613	-24.3373349245	-3.7676007302	19.9031599127	-24.2546569267	-0.8983080574
H	22.7652252194	-27.2937991978	-2.2858813607	21.944750596	-28.0365071964	-1.1895956621
H	20.0651359508	-27.188470478	-2.2611305036	19.3529240087	-27.6671183619	-1.0764871831
H	20.856789236	-27.1918965751	-4.6156853319	17.7413486554	-24.7047570812	-1.6558806395
H	19.8111130112	-26.0551078024	-5.5204034061	17.912507391	-25.5637818524	-0.0960200621
